# Decreased proportions of indoor feeding and endophily in *Anopheles gambiae s.l.* populations following the indoor residual spraying and insecticide-treated net interventions in Benin (West Africa)

**DOI:** 10.1186/1756-3305-5-262

**Published:** 2012-11-14

**Authors:** Gil Germain Padonou, Ghelus Gbedjissi, Anges Yadouleton, Roseric Azondekon, Ossé Razack, Olivier Oussou, Virgile Gnanguenon, Aikpon Rock, Michel Sezonlin, Martin Akogbeto

**Affiliations:** 1 Faculte des Sciences et Techniques de l’Universite d’Abomey Calavi, Abomey Calavi, Benin; 2 Centre de Recherche Entomologique de Cotonou (CREC), Cotonou, Benin

**Keywords:** IRS, LLITNs, Bendiocarb, Deltamethrin, Behavior, *Anopheles gambiae*, Benin

## Abstract

**Background:**

In many parts of Africa as in Benin, the main strategies of vector control are based on the scaling-up of Long Lasting Insecticide Treated Nets (LLITNs) and indoor residual spraying (IRS). The need to understand the biological implications of IRS in large scale and full coverage of LLITNs is paramount. It is in this context that the present study was conducted. It aims to evaluate the effect of a large scale IRS using a non-pyrethroid insecticide and full coverage of deltamethrin treated nets on the behavior of *An. gambiae s.l.* in the intervention areas compared to untreated areas used as controls.

**Methods:**

Mosquitoes were collected using human landing catches, pyrethrum spray catches and window exit traps to assess reduction of entry rate, endophily rate, endophagy rate and overall mortality rate in natural populations of *An. gambiae s.l.* before IRS and LLITNs intervention (2007) and after in 2008 and 2010.

**Results:**

In the IRS arm, endophily rate was 67.13% before intervention and 4.5% after intervention, whereas in the control arm it was stable at 51.67% (P > 0 .05). In the LLITN arm endophily rates also decreased after intervention. After the IRS, no gravid mosquitoes were collected from all treated localities, but LLITN performance was not that spectacular. The proportion of mosquitoes biting indoors in the IRS arm decreased from 67.09% before intervention to 42.85% after intervention, compared to a low but significant decrease (71.31% to 57. 46%) in the LLITN arm.

The use of vector control tools and behavior of the host would be the main factors that modify the behavior of taking a human blood meal observed on *An. gambiae s.l.* inside human dwellings.

**Conclusion:**

The impact on the behavior of *An. gambiae s.l.* observed with the bendiocarb used in IRS was highly effective compared with the free distribution of LLITNs in terms of mortality and the decrease of proportions of indoor feeding. Despite this efficacy, there is a need for complementary tools and research of alternative strategy oriented on effective health education, and the use of powerful tools such as IRS, LLITNs, larviciding and repellents.

## Background

Malaria remains the most important parasitic disease in Benin causing significant mortality and morbidity despite concerted efforts to control it. In many parts of Africa, the main strategies of vector control are based on the scaling-up of Long Lasting Insecticide Treated Nets (LLITNs) and indoor residual spraying (IRS) [1,2]. Many studies have shown the efficacy of indoor residual IRS and LLITNs in reducing malaria transmission and prevalence [3]. However, these methods, especially ITNs, rely on the use of pyrethroid insecticides. Unfortunately the recent evolution and spread of pyrethroid resistance in West Africa in the Mopti (M) molecular form of *Anopheles gambiae s.s* is a major concern for the sustainability of malaria prevention in Africa [4,5]. This is the reason why the research of alternate solutions using a non-pyrethroid insecticide became a priority [6]. In Zimbabwe, when bendiocarb was tested for its residual efficacy and irritation in malaria vector control, the results showed for up to 20 weeks after treatment, a mortality of 100% of *An. arabiensis* over the thatch but with a less pronounced irritant effect [7]. In another study in the Philippines, selective application of bendiocarb was very promising both in terms of efficiency and cost-effectiveness for the control of *An. flavirostris*[8]. Asidi *et al.*, have shown that the carbosulfan net gave significantly higher killing of *An. gambiae s.l.* than all pyrethroid treatments except the impregnated net with deltamethrin [9]. In Benin, a recent study [10] that included four months of evaluation of various insecticides in experimental huts, showed that Sumithion 40 WP (Fenitrothion), Master Quick ZC (mixture of chlorpyriphos 250 g/l + deltamethrin 12 g/l), and Ficam M (bendiocarb 800 g/kg), proved to be good alternatives against pyrethroid-resistant *Anopheles*. However, bendiocarb is the only product that the National Malaria Control Program (NMCP) has selected for the implementation of IRS in Benin, because the Master Quick ZC formulation is not approved by the World Health Organization Pesticide Evaluation Scheme for use. With regard to Sumithion 40 WP, doubts were raised on its safety in terms of its secondary effects and odor [10]. The choice of a control method based on IRS is a decision of Benin to reinforce the action of the LLITNs and this approach is stated in the control plan of the National Malaria Control Program. To reach this goal, two rounds of IRS of bendiocarb were carried out in the study area by the Research Triangle Institute (RTI), in collaboration with the population based on community involvement to ensure the sustainability of the strategy. IRS has been used for plateau zones (IRS arm) situated far from flooding areas, and LLITNs have been distributed to families of villages situated in flooding zones (LLITN arm) where implementation of IRS has not been adopted. The National Malaria Control Program strongly hopes to reduce malaria burden through this program. Indeed, according to results of trials sponsored by WHO in Garki in northern Nigeria [11], IRS led to a considerable decrease in the total vector population and reduction in the incidence of malaria among children, the plasmodic index and fever, and an apparent effect on mortality of 1–4 year old infants. In Kenya, IRS of fenitrothion in Kisumu [12] and the use of LLITNs in the south coast of Kenya [13], showed a decline in populations of *An. funestus s.l.* and *An. gambiae s.l.* by the IRS, while the high bed net coverage was followed by a much reduced human biting rate and a diminishing role of *An. gambiae s.s*. in malaria transmission. The monitoring of behavioral responses of mosquitoes to insecticides is critical to the understanding of how chemicals function in the control of disease transmission [14]. As the international community has now prioritized national and regional elimination with a long-term ultimate goal of malaria eradication [15], the need to understand the biological implications of IRS in large scale and full coverage of LLITNs is paramount. After the implementation of the IRS in Benin by the NMCP, it is important to understand its impact on the behavior of *An. gambiae s.l.* in contact with walls treated with insecticide. In this context, the present study aims to evaluate the effect of a large scale IRS using a non-pyrethroid insecticide and full coverage of deltamethrin treated nets on the behavior of *An. gambiae s.l.*in the intervention areas compared to untreated areas used as controls.

## Methods

### Study area

**Figure 1:**
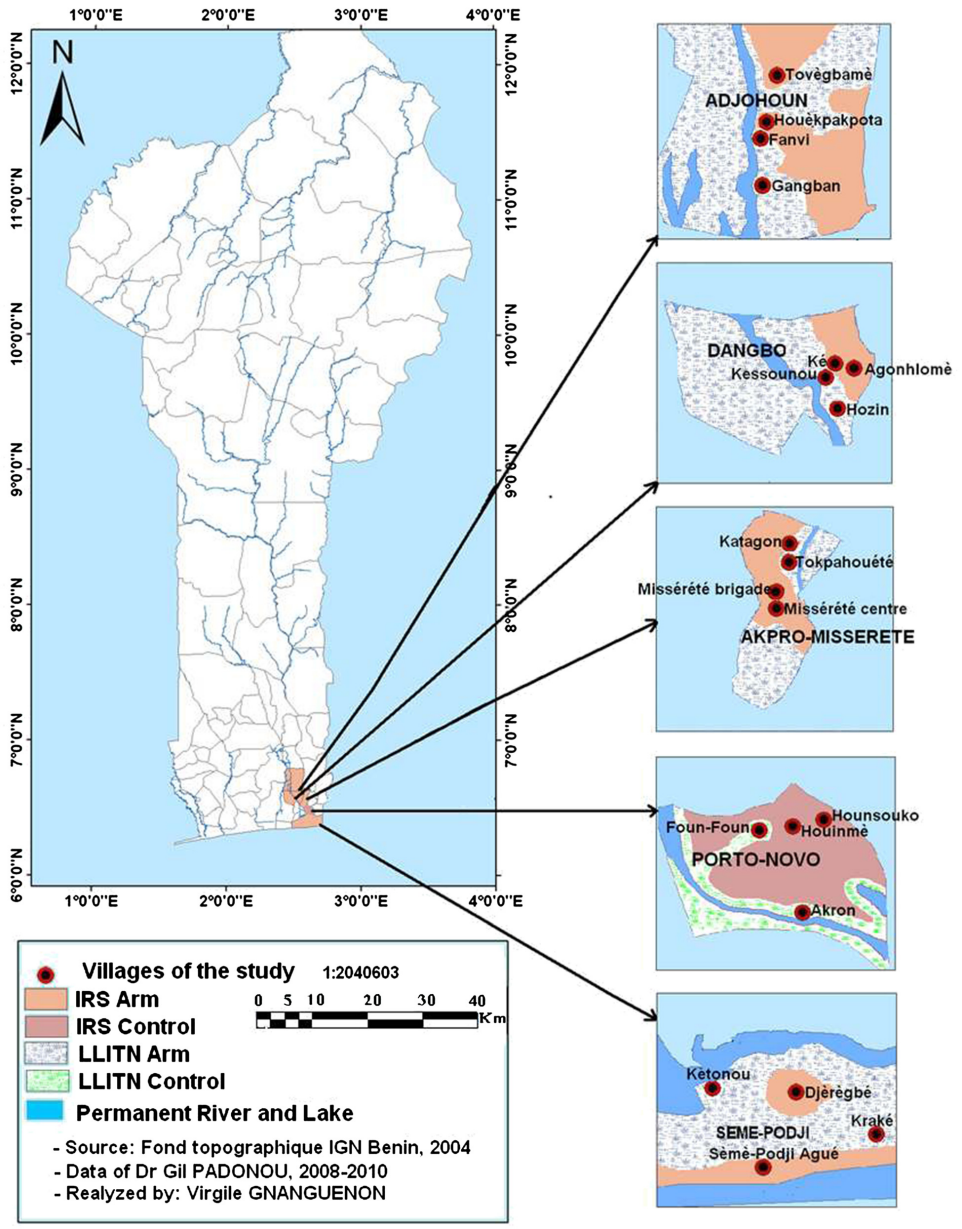
Map of the study area.

The study was conducted in 4 districts of Oueme region in South-East of Benin (Figure 1): Adjohoun, Dangbo, and Seme-Misserete Kpodji which are retained by the health authorities for the first indoor residual spraying campaign in Benin. The four districts cover an area of 977 km^2^ and an estimated 64,799 households. There are 62,890 children aged <5 years in 174 villages [16]. From 2002 to 2006, Oueme was the region with the highest rates of malaria-associated mortality [16]. The region is characterized by a sub-equatorial type climate. Our study also included Porto-Novo, which served as a control because it has the same ecological and geographic characteristics as the four districts mentioned above. Porto-Novo is the administrative capital of Benin, also in Oueme region (Figure 1). Oueme region is characterized by the presence of two types of environment. The first environment is a plateau zone situated far from flooding areas. In the plateau area, mosquito breeding sites are created particularly during the rainy seasons; more than 90% of households have been treated with bendiocarb at a dose of 400 mg/m^2^. The second environment is the peripheral area represented by a swampy zone on the border of the Oueme River and Lake Nokoue. This peripheral area is made up of marshy land converted to vegetable gardens. Land management in this vegetable growing area creates a perfect breeding site for *An. gambiae s.l.*, the main vector of malaria, which is highly resistant to pyrethroids [16]. In the present study, the plateau zone is referred to as the “IRS arm”, and the swampy zone is called “LLITN arm”. In the swampy zone, IRS was not implemented because of the presence of the two bodies of water, which could be at risk of contamination by insecticides. Therefore, LLITNs were distributed to these households in this area, and particular attention was given to children less than five years of age and pregnant women [17]. An estimated distance between 5 and 7 km separated the plateau and the flood areas [18]. This distance was sufficient enough to prevent migration of mosquitoes from one area to another. The houses in both areas are generally built in a similar shape. These houses are made of either mud or cement with large eave gaps facilitating entry and exit of mosquitoes [16]. However, the human population density is high in both areas, and mosquitoes do not need to travel far to feed [17].

### Indoor residual spraying of bendiocarb in the plateau area and LLITNs distribution in the swampy area

Two rounds of IRS of bendiocarb were carried out in the plateau areas (IRS arm) of districts of Adjohoun, Dangbo, Misserete and Seme. The first round was carried out in July 2008 and the second, eight months later, in March 2009. Indeed, in a recent study, this insecticide appeared especially efficient in phase II evaluation against malaria vectors [17]. The two applications were completed by volunteers selected from the local community and trained by the RTI team, the implementing partner of the U.S. Agency for International Development. According to RTI, the coverage rate was more than 90% for each of the two rounds. In the swampy areas a total of 48,819 LLITNs (Permanet 2.0; Vestergaard Frandsen, Lausanne, Switzerland) were distributed in October 2008 and May 2009. They were distributed to 47,524 households. According to RTI, more than 90% of children less than five years of age and pregnant women received LLITNs.

### Mosquito sampling and identification

The sampling was carried out in 4 villages (2 in the plateau area and 2 in the peripheral area) per district by using indoor and outdoor human landing catches (HLC) to identify the changes in biting behaviour of mosquitoes induced by the presence of bendiocarb on the walls or deltamethrin in the fiber of PermaNets. The sampling was carried out every month for two consecutive nights per survey (8 person-night per district per survey). Catches were conducted between 21.00 and 05.00 hrs. Teams of collectors were rotated among the collection points on different collection nights to minimize sampling bias. Informed consent from all volunteers was obtained before their participation in the study.

Human landing catches were carried out during January 2008–December 2009 and spanned two rounds of IRS. To measure the impact of the intervention on the endophagy rate, we compared the values indicated for the same periods, January–July 2008 before intervention and January–July 2009 after intervention. We excluded August–December 2008 and 2009 from the analysis because these two periods were those of IRS implementation, but there is no available database for these periods for before and after intervention.

In addition, to evaluate the impact of interventions on reduction of entry rate, endophily rate and mortality rate induced by the presence of insecticides, 4 bedrooms were selected in each intervention area. Mosquitoes were collected by using window exit traps. The collections of live mosquitoes were carried out using a mouth aspirator and transferred into holding tubes and provided with cotton wool wetted with a 10% honey solution to record delayed mortality after 24 h. Dead mosquitoes collected in the trap and on the floor inside the bedroom were transferred into plastic cups to be identified and used to determine the immediate mortality. Furthermore, in the meantime, morning catches within the same bedrooms were undertaken using pyrethrum spray catches.

The sampling of mosquitoes was carried out in the same bedroom and at the same frequency during the intervention periods before and after.

All anophelines were sorted and assigned to species based on morphological characters using standard identification keys [19]. The female mosquitoes belonging to *An. gambiae s.l.*were classified according to the state of their abdomens (unfed, partially fed, fully fed or gravid) [20].

### Parameters measured

Reduction of entry rate is the difference of the number of *An. gambiae s.l.* captured after intervention multiplied by 100 and divided by the number caught before intervention in the same bedroom.

Endophily rate is the percentage of the number of *An. gambiae s.l.* at rest divided by the total number collected by indoor residual spraying and the window trap.

Endophagy rate is the percentage of the number of indoor bites divided by the total number of indoor and outdoor bites.

Overall mortality rate is immediate mortality rate + delayed mortality rate recorded after 24 h.

### Statistical analysis

Data were analyzed using SPSS version 16.0 (SPSS Inc., Chicago, IL). The efficiency of the intervention was evaluated by using the Kruskal-Wallis test to compare parameters (blood feeding rate, gravidity rate, endophily rate) between the periods before and after intervention. The Fisher exact test was used to compare mortality rates between these two periods. The significance level was set at 5%.

### Ethical approval

This study received the approval of the Ministry of Health and the Centre for Entomological Research of Cotonou (CREC). The voluntary mosquito collectors gave their consent before participating in the study. Malaria prevention and curative treatments were provided to all sleepers according to World Health Organization (WHO) recommended regimen on the basis of fever and detectable *P. falciparum* parasitemia. They were all vaccinated against yellow fever.

## Results

### Decrease of entry rate of *An. gambiae s.l.* after interventions

Before IRS interventions during May to July 2008 a total of 928 *An.gambiae* were collected by exit window traps and PSC, for all localities Adjohoun, Dangbo, Misserete_1_, Misserete_2_ and Seme. But after IRS interventions, this number had fallen drastically to 89, representing a reduction of 90.40% over the same period. Despite a lower irritancy of bendiocarb, it induced a strong repellent effect on the behavior of *An. gambiae s.l.* after IRS. In many localities the reduction of *An. gambiae s.l.* entry rate, was very significant (Table 1), particularly in Seme IRS arm where no *An. gambiae s.l.* were caught by exit window traps and PSC. While in the untreated control IRS arm, there was an opposite trend (Table 1): the number of *An. gambiae s.l.* increased by a proportion of 50%. But in the LLITN arm there was a reduction of entry rate in Adjohoun (20.88%), whereas in Dangbo and Seme there was an increase of entry rate of mosquitoes in the respective proportions of 21.95% and 14%.

**Table 1:** **Reduction of entry rate of *Anopheles gambiae s.l. *observed before and after two interventions in districts of Adjohoun, Dangbo, Misserete and Seme**

**Districts**	**Number of females caught before intervention (May-July 2008)**	**Number of females caught after intervention (May-July 2009)**	**Reduction of entry rate %**
**IRS arm**			
Adjohoun	84	32	61.90
Dangbo	84	48	42.85
Misserete _1_	84	5	94.04
Misserete _2_	288	4	98.61
Seme	388	0	100
Akron Control	88	132	−50
**LLITN arm**			
Adjohoun	91	72	20.88
Dangbo	82	100	−21.95
Seme	90	216	−140
Akron Control	404	384	4.95

### Decrease of endophily rate

During the period before intervention, from a total of 928 *An. gambiae s.l.* captured by window exit traps and PSC, 623 were endophilic (67.13%) in the IRS arm. But after IRS interventions, only 4 *An. gambiae s.l.* were caught in the bedrooms in Dangbo from a total of 89 *An. gambiae s.l.*, whereas in the control the endophily rate was stable at 51.67% (P > 0 .05) (Table 2). In peripheral areas of Adjohoun, Dangbo and Seme where LLITNs were distributed, endophily rates also decreased, respectively to 11.11% (8/72), 52% (52/100), 32.50% (74/216) after LLITNs interventions. But in Dangbo the decrease was not significant (P > 0 .05) (Table 2). In the control LLITN arm, the variation of endophily was not significant between the two periods before (53.33%) and after (52.08%) intervention (P > 0 .05). When comparing the impact of IRS and LLITN in the same districts in terms of reduction of *An. gambiae s.l.* endophily rate, the decrease due to the IRS (100%) was higher than the decrease due to LLITN (43.95%) in the district of Seme. This has been observed less significantly in Adjohoun and Dangbo.

**Table 2:** **Endophily rate of *Anopheles gambiae s.l. *observed before and after two interventions in districts of Adjohoun, Dangbo, Misserete and Seme**

**Districts**	**Before intervention (May-July 2008)**				**After intervention (May-July 2009)**			
	**Number of females caught**	**Number of *An. gambiae* caught by PSC**	**Endophily rate**		**Number of females caught**	**Number of *An. gambiae* caught by PSC**	**Endophily rate**	
			**Mean**	**Confidence interval**			**Mean**	**Confidence interval**
**IRS arm**								
Adjohoun	84	58	68.83^a^	[58.02-78.69]	32	0	0^b^	[0.00-10.91]
Dangbo	84	53	63.33^a^	[51.87-73.37]	48	4	8. 33^b^	[2.32-19.98]
Misserete _1_	84	52	61.83^a^	[50.66-72.29]	5	0	0^b^	[0.00-52.20]
Misserete _2_	288	210	73^a^	[67.39-77.97]	4	0	0^b^	[0.00-60.25]
Seme	388	250	63.67^a^	[59.44-69.20]	0	0	-	-
Akron Control	88	45	49.33^a^	[40.24-61.95]	132	68	51.67^a^	[42.66-60.30]
**LLITN arm**								
Adjohoun	91	55	60.50^a^	[49.64-70.54]	72	8	11.11^b^	[4.92-20.73]
Dangbo	82	50	61.50^a^	[49.57-71.57]	100	52	52^a^	[41.78-62.10]
Seme	90	55	60.83^a^	[50.25-71.21]	216	74	32.50^b^	[27.95-41.00]
Akron Control	404	216	53.33^a^	[48.47-58.42]	384	200	52.08^a^	[46.96-57.18]

For the mean of each district, values of the same line which carry same letters in exposant were not significantly different (p > 0.05).

### Gravidity rate of *An. gambiae s.l*

Before intervention, 348 *An. gambiae s.l.* from a total of 928 (37.5%) were gravid in the IRS arm. Misserete_2_ and Adjohoun had the highest (41.6%) and lowest (30.16%) rates. After the IRS no gravid mosquitoes had been collected in all localities (Table 3). Compared to IRS, LLITN performance was not as spectacular (Table 3). The rate of gravidity fell to 31.08% and 24.98% respectively in Adjohoun and Dangbo, whereas it increased to 25.01% in Seme. The gravidity rate in the control has also decreased from 2.5% in the IRS arm and 14.17% in the LLITN arm (Table 3).

**Table 3:** **Gravidity rate of *Anopheles gambiae s.l. *observed before and after IRS and LLIN intervention in districts of Adjohoun, Dangbo, Misserete and Seme**

**Districts**	**Before intervention (May-July 2008)**				**After intervention (May-July 2009)**			
	**Number of females caught**	**Number of gravid mosquitoes**	**Gravidity rate**		**Number of females caught**	**Number of gravid mosquitoes**	**Gravidity rate**	
			**Mean**	**Confidence interval**			**Mean**	**Confidence interval**
**IRS arm**								
Adjohoun	84	26	30.16^a^	[21.31-41.98]	32	0	0^b^	[0.00-10.91]
Dangbo	84	31	36^a^	[26.63-48.13]	48	0	0^b^	[0.00-7.42]
Misserete _1_	84	29	33.66^a^	[24.48-45.70]	5	0	0^b^	[0.00-52.20]
Misserete _2_	288	120	41.16^a^	[35.91-47.60]	4	0	0^b^	[0.00-60.25]
Seme	388	142	36.16^a^	[31.79-41.61]	0	-	-	-
Akron Control	88	35	41^a^	[29.49-50.77]	132	53	40^a^	[31.72-49.04]
**LLITN arm**								
Adjohoun	91	41	44.33^a^	[34.60-55.85]	72	22	30.55^b^	[20.24-42.53]
Dangbo	82	38	46.66^a^	[35.25-57.70]	100	35	35^b^	[25.73-45.19]
Seme	90	35	38.66^a^	[28.79-49.75]	216	101	48.33^a^	[39.96-53.65]
Akron Control	404	176	42.33^a^	[38.67-48.56]	384	133	36.33^a^	[29.88-39.63]

For the mean of each district, values of the same line which carry same letters in exposant were not significantly different (p > 0.05).

### Decrease of endophagy rate

**Figure 2:**
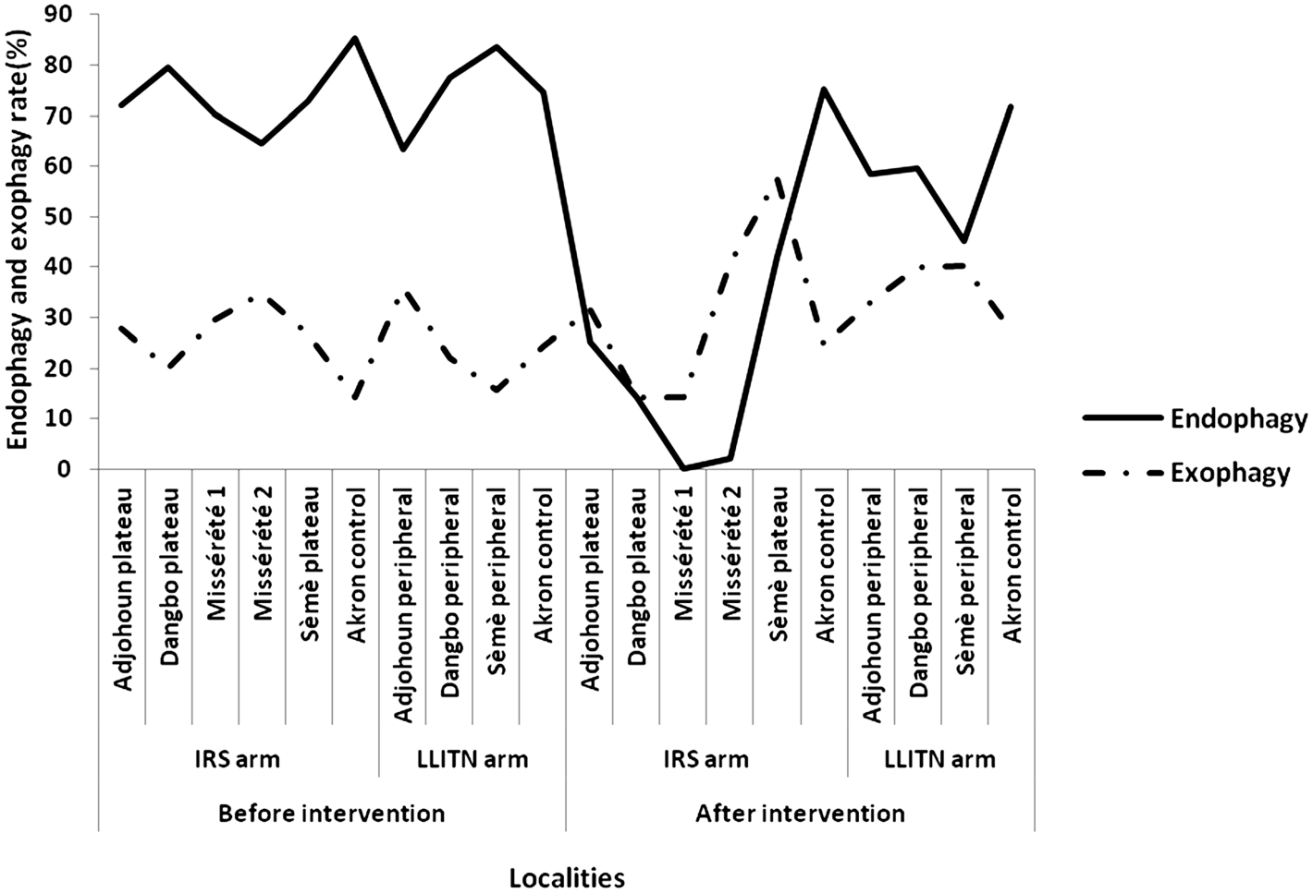
Graphical variation of the proportion biting indoors (Endophagy) and that of the ones biting outdoors (Exophagy) in the IRS arm and the LLITN arm during the periods before and after interventions.

Before intervention *An. gambiae s.l.* was endophagic in all localities (Figure 2). The proportion biting indoors were 85.43%, 72.14%, 79.57%, 70.43% and 64.43% in Porto Novo, Adjohoun, Dangbo, Misserete _1_, Misserete _2_ and Seme respectively (Figure 2). But after IRS intervention *An. gambiae s.l.* tends to take a blood meal preferably on catchers installed outside rooms in the IRS arm (Figure 2). Indeed, the proportion biting indoors in the IRS arm significantly (p < 0.05) decreased from 67.09% before intervention to 42.85% after intervention (24.24% of reduction), compared to a low but significant decrease (71.31% to 57. 46%) in LLITN arm. This was not the case in the control area (p > 0.05) during the period after intervention.

### Mortality rate of *An. gambiae s.l*

We have noticed that all live mosquitoes collected in window traps and transferred into plastic cups were dead before 24 hours had elapsed. Therefore, we have considered them as mosquitoes immediately dead, including all dead mosquitoes collected from floors and in the exit window traps. In the IRS area before intervention the mortality rate was almost zero (4/928 *An. gambiae s.l.* were dead) (Table 4). However, after the IRS, the lethal effect of bendiocarb was very significant, with mortality rates of 64.58%, 80% and 75% respectively observed in Dangbo, Misserete _1_ and Misserete _2_ (Table 4). In Adjohoun this rate was lower (37.5%), whereas it had been 0% in the control area (p > 0.05) (Table 3). However, after distribution of LLITNs, the mortality rate increased respectively to 8.33%, 23% and 13.88% in localities of Adjohoun, Dangbo and Seme. Compared to the lethal effect of bendiocarb in IRS areas, LLITNs are proven less effective.

**Table 4:** **Mortality rate of *Anopheles gambiae s.l. *observed before and after IRS and LLITN interventions in districts of Adjohoun, Dangbo, Misserete and Seme**

**Districts**	**Before intervention (May-July 2008)**				**After intervention (May-July 2009)**			
	**Number of females caught**	**Number of dead mosquitoes**	**Mortality rate**		**Number of females caught**	**Number of dead mosquitoes**	**Mortality rate**	
			**Mean**	**Confidence Interval**			**Mean**	**Confidence interval**
**IRS arm**								
Adjohoun	84	2	**2.3**^**a**^	[0.29-8.34]	32	12	**37.5**^**b**^	[21.10-56.31]
Dangbo	84	1	**1.04**^**a**^	[0.03-6.46]	48	31	**64.58**^**b**^	[49.45-77.84]
Misserete _1_	84	1	**1.11**^**a**^	[0.03-6.46]	5	4	**80**^**b**^	[28.36-99.50]
Misserete _2_	288	0	**0**^**a**^	[0.00-1.27]	4	3	**75**^**b**^	[19.41-99.37]
Seme	388	0	**0**^**a**^	[0.00-0.95]	0	-	**-**	-
Akron Control	88	1	**0.83**^**a**^	[0.03-6.17]	132	0	**0**^**a**^	[0.00-2.76]
**LLITN arm**								
Adjohoun	91	0	**0**^**a**^	[0.00-3.98]	72	6	**9.72**^**b**^	[3.12-17.26]
Dangbo	82	0	**0**^**a**^	[0.00-4.40]	100	23	**25.69**^**b**^	[15.17-32.49]
Seme	90	1	**0.16**^**a**^	[0.03-6.04]	216	30	**14.13**^**b**^	[09.57-19.23]
Akron Control	404	0	**0**^**a**^	[0.00-0.91]	384	0	**0**^**a**^	[0.00-0.96]

For the mean of each district, values of the same line which carry same letters in exposant were not significantly different (p > 0.05).

## Discussion

The high decrease of entry rate (42.85 à 100%) of *An. gambiae s.l.* natural populations into bedrooms, could be explained by the large scale of IRS with bendiocarb that created a stressful environment, which in turn could lead to impressive reduction of entry rate of mosquitoes. This is consistent with previous studies that showed that the unpleasant atmosphere created by the presence of bendiocarb on the walls inside houses is harmful to the mosquitoes and leads to an increase in the exit rate [17]. Conversely, the strong irritant effects that we observed during the evaluation in experimental huts [10] for deltamethrin, showed reduction of entry rate was less effective in LLITNs areas. This difference in efficacy could be due to the mass community effect of bendiocarb used on a large scale. Indeed, in IRS areas, 2,623 kg of bendiocarb were sprayed in 142,814 bedrooms during the first intervention and 2,751 kg in 156,233 bedrooms during the second intervention; 90 to 100% of bedrooms were treated, according to RTI (Research Triangle International) who carried out the spraying operation. This is consistent with previous studies that showed that community-wide use of insecticide-treated bednets (ITBN) engenders a mass effect [21]. The data analyzed on a cohort of children, revealed for those not using ITBNs, an increasing level of ITBN usage within the area surrounding each child was associated with a decreased risk of developing malaria. This effect was significant in areas at distances of up to 1.5 km away from each child [21]. In addition, some beneficiaries of LLITNs do not use them, but continue to use the untreated nets they had before. Prior to free distribution of LLITNs, it was shown that heat, choking, beliefs and taboos seem to be barriers to the use of bednets in the study area [16]. It is also possibly due to their poor living standards. People sell LLITNs to address other problems, as was the case in trials sponsored by The WHO in the Congo and Tanzania [22]. For LLITNs to be fully effective, requires that community members are actively involved in the process, to ensure that nets are used even during seasons when such use is unpleasant because of the heat and insect bites do not seem numerous enough to justify it [22].

The endophily rate of *An. gambiae s.l.* observed before the IRS and LLITN interventions corroborates previous reports of anopheline behaviour [23,24]. However, after the IRS and LLITN interventions a dramatic decrease of the endophily rate was observed in IRS area. This could be explained by a strong decrease in the proportion of gravid and half gravid mosquitoes, according to Table 3. The unpleasant atmosphere created by the presence of bendiocarb on the walls inside houses was harmful to the mosquitoes and might be the cause of this shift in behavior. Furthermore, the impressive reduction of entry rate of *An. gambiae s.l.* could justify this shift. Despite this deterrent effect a low proportion of *An. gambiae s.l.* enter bedrooms. But once on the walls, they absorb the bendiocarb which kills them and they do not have time to bite and to rest inside to digest their blood meal. Indeed, other studies previously conducted [10,25,26] have shown the effectiveness of alternative insecticides such as carbamates to control *An. gambiae s.l.* resistant to pyrethroids. Furthermore, the present study confirms the absence of *An. gambiae s.l.* resistance to bendiocarb in southern Benin [16,27]. Conversely, *An. gambiae s.l.* resistance to pyrethroids [18,25,28] was corroborated and could justify the difference in efficacy of LLITNs impregnated with deltamethrin compared to that using IRS based on bendiocarb.

The findings have also demonstrated that large scale IRS can alter *An. gambiae s.l.* populations and reduce the epidemiological importance of indoor-biting mosquitoes. This decrease was also observed in the LLITNs area, but in a lower proportion. This is consistent with others studies showing that IRS [17] and ITNs in Somalia [29] and Tanzania [30] can reduce the mean density, survival, infectiousness and fitness of mosquito populations.

Conversely, in the control area *An. gambiae s.l.* remained endophagic in the period after intervention, whereas it has been more exophagic in intervention areas. In this context, the human-biting behaviour of vectors in Oueme region appears to be independent of population density for these species [31]. Nevertheless, a very plausible case [32] is presented that correlates community-wide ITN use with significant changes in the biting profile of the principal malaria vectors. This indicates that factors relating to locality and seasonal climatic variations would have little effect on shifts of behaviour of taking human blood.

The use of vector control tools and behaviors of the host would be the main factors that modify the behavior of sucking human blood observed on *An. gambiae s.l.*. Indeed recent studies [33,34] showed that the long-term indoor application of residual insecticides contributes towards an increased tendency for outdoor feeding among malaria vector populations. This is expected to erode the efficacy of malaria vector control interventions over time, much as increased insecticide resistance would [35].

Despite the effectiveness of bendiocarb used for IRS, it has the disadvantage of having a short residual effect [10,36]. In this case it appears that the LLITNs, although providing modest efficacy against pyrethroid resistant *An. gambiae s.l.*, are necessary to supplement IRS because of the long duration of the action of deltamethrin and the role as a mechanical barrier played by this tool [17,22], against mosquitoes. Regardless of a shift in host seeking behaviour of *An. gambiae s.l.*, other possibilities for outdoor anti-vector interventions need to be explored, in combination with ongoing IRS and LLITN distribution because of the short residual effect mentioned above.

## Conclusion

The impact on the behaviour of *An. gambiae s.l.* observed with the bendiocarb used in IRS was highly effective compared with the free distribution of LLITNs in terms of mortality and the decrease of proportions of indoor feeding. However, this decrease is not enough to prevent a new infection of *Plasmodium falciparum* by outdoor biting mosquitoes. In this case it would be advisable for people to go to bed early and to avoid infective bites outdoors. Therefore, the personal protection and collective protection respectively conferred by the IRS and LLITN are not enough to eradicate malaria. There is therefore a need for complementary tools [37,38], and research of alternative strategies oriented on effective health education, people’s empowerment and participation, and the use of powerful tools like IRS, LLITNs, larvicides and repellents.

## Competing interests

The authors declare that they have no competing interests.

## Authors’ contributions

GGP, GG, VG, RO and MA designed the study. GGP, RA and OO carried out the experiments. GGP and GG analyzed the data. GGP, GG and AY drafted the manuscript. GGP, MA, GG, AY, RA and MS critically revised the manuscript. All authors read and approved the final manuscript.
